# A comparative evaluation between dermatoglyphic patterns and different terminal planes in primary dentition

**DOI:** 10.4317/jced.55259

**Published:** 2018-12-01

**Authors:** Vignesh Ravindra, Vishnu Rekha, Sankar Annamalai, Ditto Sharmin, Parisa Norouzi-Baghkomeh

**Affiliations:** 1 MDS. Senior Lecturer. Department of Pedodontics and Preventive Dentistry. Saveetha Dental College and Hospital. Chennai

## Abstract

**Background:**

To assess the correlation between different dermatoglyphic patterns with the terminal planes in deciduous dentition.

**Material and Methods:**

300 children who are 3-6 years old with complete primary dentition were recruited and the pattern of molar terminal plane was recorded in the proforma. Finger prints of the distal phalanges of these subjects were recorded using ink and roller method and were analysed for the finger print pattern by a forensic specialist. The pattern were classified based on classification given by Galton. The finger ridge counts were also measured.

**Results:**

Ulnar loop pattern was the most predominant dermatoglyphic pattern. Absence of arch pattern in ring and little fingers of left hand and higher ridge count in left little finger when compared to the right hand were related to Mesial step. Presence of whorl pattern in both right and left middle finger and higher total finger ridge count in left hand when compared to the right hand were related to distal step. Flush terminal plane was related to absence of arc pattern in ring finger of left hand.

**Conclusions:**

Dermatoglyphics can be used as a non invasive analytical tool to predict the terminal plane in primary dentition.

** Key words:**Dermatoglyphics, terminal planes, primary dentition.

## Introduction

The terminal plane of the second molars in the primary dentition have a significant role in determining the occlusion of the succedaneous dentition (1,2). The characteristics of occlusion in permanent dentition can be predicted based on the features of child’s dentoalveolar system during the formative years (3). Though the primary dentition provides the framework and foundation for proper eruption and alignment of the permanent dentition, those characteristic features vary among different ethnic groups of population (4-6). Dermatoglyphics, which is the study of skin carvings, have come a long way from astrological view to become a tool to predict various dental anomalies. Although its link with occlusion in permanent dentition is slowly getting established (7-13), the association with primary dentition is not yet studied. So this study was aimed to assess the possible correlation between dermatoglyphics and the different terminal planes in primary dentition.

## Material and Methods

This study was conducted among 300 children aged 3-6 years attending the out-patient department of the Department of Pediatric and Preventive Dentistry. Ethical clearance was obtained from Institutional Review Board. Study purpose and procedures were explained to the parents and only those who gave a consent to participate were included in the study. Children with completely erupted primary dentition before initiation of first transitional period were included in the study. Uncooperative children, previous history of orthodontic treatment, previous history of burn or chemical injury or lesions on distal phalanges of hands, children with grossly decayed teeth or proximal caries or premature extraction of primary teeth affecting the molar relation and different molar relationships on either side of the same subject were excluded from the study. Children were taught multiple times to bite in centric occlusion and two calibrated examiners were trained to assess the molar relationships based on the classification given by Baume (1950) as mesial step, distal step and flush terminal plane (14). The assessment was done using a mouth mirror and recorded in the proforma. A total of 100 children were taken for each molar relationship to standardize the number of children under each group.

**Figure 1:**
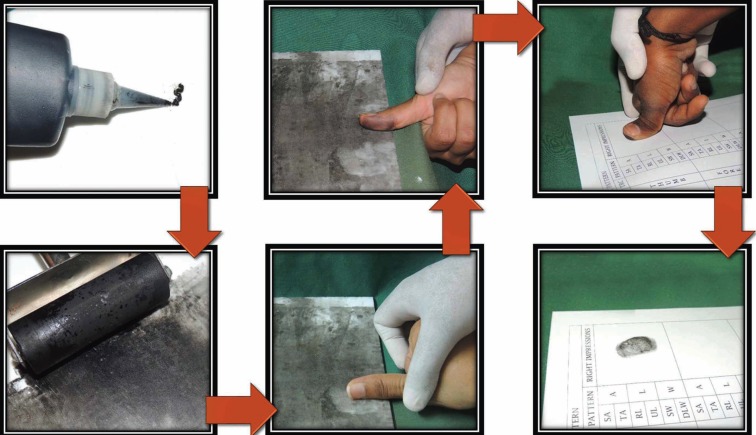
The process used in the study to record dermatoglyphic pattern.

The ink and roller method, suggested by Cummins and Midlo (15), was preferred to record the finger prints. Children were asked to wash their hands using soap to remove any dirt and sebaceous secretions on the palms. A small amount of Black printer’s ink was dispensed on the inking slab and was evenly spread to a thin dull finish using a roller. The bulb of each distal phalange of the digits in both hands were placed at right angles to the inking slab and rolled over the ink until the bulb faced opposite side. Children were asked to transfer the finger print to a bonded white paper by rolling in the same manner with minimal pressure (Fig. 1). Each print was checked for clarity and if any smudging of the print were noticed, the print was repeated once again. The collected finger prints were analysed using a magnifying glass by a forensic specialist who was trained to analyse the prints. The analyst was blinded about the age, gender and molar relation of the children. The analyst read the finger prints based on the basic classification given by Galton (1892) (16) as arch, loop and whorl and further subclassified as simple arch, tented arch, ulnar loop, radial loop, simple whorl, double loop whorl and central pocket whorl.

The total finger ridge count was calculated based on the method given by Cummins and Midlo (15). The approximate center of each pattern (core) and corresponding confluence of three ridge systems that form angles of approximately 120° with one another (triradii) were defined. A straight line was drawn passing through these two points. The ridge count was calculated by counting the number of ridges cut by this line. In this study, the largest of the two ridge counts of each finger was taken as the finger ridge count for that finger. The finger ridge counts were summed for each hand separately and for both hands together to obtain the total finger ridge count.

-Statistical analysis

The data values were tabulated and subjected to statistical analysis. Chi-Square test was applied to compare proportions between all the groups and also for gender comparison. Fisher’s exact test was used when any expected cell frequency of less than five were obtained. Paired T-Test and McNemar’s test were applied to compare values between right and left hand. SPSS version 22.0 was used to analyse the data. A *p*-value of <0.05 is considered as statistically significant.

## Results

The mean age group of children was 4.99 ± 0.67 years. Among the children having mesial step, 39% were males and 61% were females. For the children having distal step, 45% were males and 55% were females. In children having flush terminal plane, 57% were males and 43% were females.

Ulnar loop pattern was the most predominant pattern which was equally distributed in all the children. In mesial step, a statistically significant decrease in arch pattern in the ring finger (*p*=0.023) and in the little finger (*p*=0.008) of left hand were noted. In specific patterns, a significant increase in ulnar loop pattern in the ring finger (*p*=0.032) and absence of simple arch pattern in little finger of the left hand was found to be significant (*p*=0.007). In the right hand, a reduction in arch pattern and an increase in loop pattern in the middle and little fingers were noticed in right hand, which were not statistically significant (*p*=0.557 and 0.098 respectively). In specific patterns, a significant increase in ulnar loop pattern and reduction in simple arch pattern was noticed in the right little finger (*p*=0.011). For distal step, a significant increase in whorl pattern in the ring finger (*p*= 0.023) and loop pattern in the little finger (*p*=0.008) were found in left hand. An increase in simple whorl pattern in the left ring finger and ulnar loop pattern in left little finger was noticed, which were statistically significant (*p*=0.032 and 0.007 respectively). In the right hand there was an increase in whorl pattern in the ring finger, which was not statistically significant (*p*=0.436). An increase in ulnar loop pattern and tented arch pattern in little finger were noted in the right hand, which were statistically significant (*p*=0.011). In flush terminal plane, a significant reduction in arch pattern in the ring finger (*p*=0.023) and a significant rise in loop pattern in the little finger (*p*=0.008) were noticed in left hand while the right hand showed an increase in whorl pattern in the ring finger that was not significant (*p*=0.436). For specific patterns, a significant increase in simple whorl pattern and absence of tented arch pattern in ring finger of the left hand (*p*=0.032). There was also an increase in ulnar loop pattern in little finger of left hand was statistically significant (*p*=0.007). An increase in ulnar loop pattern and absence of tented arch pattern in little finger of right hand were noticed which were statistically significant (*p*=0.011). The significant relations are provided in  Table 1.

**Table 1:**
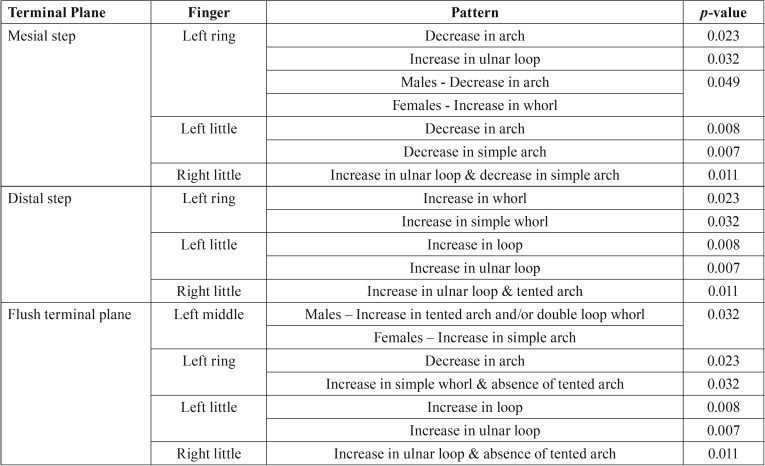
Significant correlations in the patterns for each terminal planes.

Comparison between the left and right hands showed a combination of absence of whorl pattern in left middle finger and arch pattern in right middle finger or combined absence of arch pattern in left middle finger and whorl pattern in right middle finger was significant (*p*=0.005) in children with mesial step. Presence of arch pattern in both right and left little finger was also significant (*p*=0.047) in children with mesial step. Presence of whorl pattern in both right and left middle finger was significant (*p*<0.001) in children with distal step. Presence of loop pattern or whorl pattern in both left and right index finger was statistically significant (*p*=0.009) in children with flush terminal plane. There were no significant differences among the specific patterns between different terminal planes when compared between the left and right hands. The significant relations are provided in  Table 2.

**Table 2:**
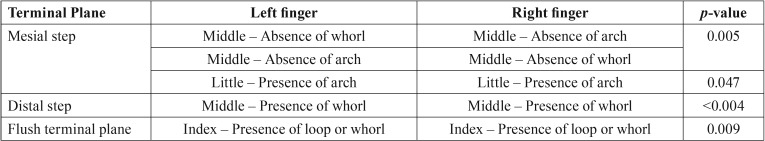
Significant correlations on comparison of patterns between left and right hands for each terminal planes.

On comparison based on gender, significant values were noticed only with mesial step where a complete absence of arch pattern in males and increased whorl pattern in females in left ring finger were noticed (*p*=0.049). Based on specific patterns, significant values were noticed only with flush terminal plane where increased tented arch pattern and/or double loop whorl pattern in males and increased simple arch pattern in females in left middle finger were noticed (*p*=0.032). The significant relations are provided in  Table 1.

The mean total finger ridge count in the left hand, right hand and both hands showed significantly lesser count in mesial step when compared with other terminal planes (*p*<0.001 for all the three comparisons). The left hand showed a significant reduction in finger ridge count in thumb and ring finger for mesial step (*p*<0.001). It was higher in the left middle finger for distal step, which was also statistically significant (*p*<0.001). In the right hand, a significant increase in finger ridge count was noticed in fore and little finger for flush terminal plane (*p*=0.042 & 0.003 respectively) and in middle finger for distal step (*p*<0.001). Lower ridge count was noticed in the right thumb and ring finger for mesial step which was statistically significant (*p*<0.001).

On comparison between the hands, children with mesial step had a higher ridge count in left little finger when compared to the right (*p*=0.007) and children with distal step showed higher total finger ridge count in left hand than right hand, which was statistically significant (*p*=0.045). No statistically significant values were obtained when comparing the total finger ridge count between males and females. The significant relations are provided in  Table 3.

**Table 3:**
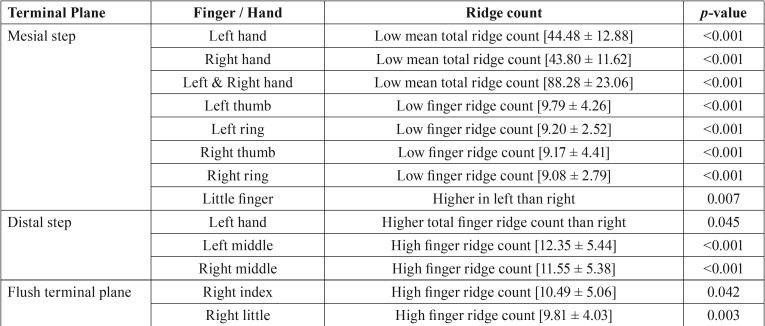
Significant correlations in the ridge counts for each terminal planes.

## Discussion

Every human is unique and distinct in that they exhibit their own characteristic pattern. These unique patterns can be exhibited commonly as dermal ridges in the palm and distal digits of hands and feet. These were commonly used in the field of forensic dentistry for individual identification as “Dermatoglyphics”. The term comes from two Greek words derma meaning skin; glyphe meaning carve (17). As defined by Cummins and Midlo in 1929, it refers to the study of the intricate dermal ridge configuration on the skin covering, the palmar and plantar surfaces of the hands and feet (18). Dermatoglyphics has been reported to be associated with a number of physiological and pathological conditions in the oral cavity: dental caries, cleft lip and palate, periodontal diseases, oral carcinomas, bruxism, malocclusions etc (19,20).

The development of occlusion is a result of combination of genetic and environmental factors. The effect of a particular environmental factor on phenotype varies depending on genetic background, which ultimately determines facial and dental morphology (21). A proper understanding of dermatoglyphics and dental structures can only be obtained “with knowledge on their phylogenetic and ontogenetic histories”. It is known that any factor active during the time period of genetic expression, is bound to affect all structures developing at that time (9). The epidermal ridges of the fingers and palm and the facial structures like lip, alveolus and palate originate during the same embryonic period concurrently (22) from the same embryonic tissue, i.e. the ectoderm. Thus genome in the genetic message is deciphered during this period and is reflected in dermatoglyphic patterns.

From literature search, it was found that this is the first study to be done in children with complete primary dentition. The study results helped us to predict a few patterns which were related to specific molar relationships. In our study, among dermatoglyphic patterns, ulnar loop pattern was found to be equally distributed in all the children. This was in accordance with BR Reddy *et al.* (9), Eslami *et al.* (23) and Deepti *et al.* (24) studies who had reported the same predominance in permanent dentition.

The current study showed that children with Mesial step showed absence of arch pattern in ring and little fingers of left hand; combined absence of whorl pattern in left middle finger and arch pattern in right middle finger or vice-versa; complete absence of arch pattern in males and increased whorl pattern in females in left ring finger; presence of ulnar loop pattern in left ring finger and right little finger; absence of simple arch in left little finger and simple whorl in right little finger; reduced finger ridge count in thumb and ring finger of both hands; higher ridge count in left little finger when compared to the right hand; and total finger ridge counts were lower in both the hands; individually and together. Children with Distal step showed presence of whorl pattern and/or loop pattern in ring and little fingers of left hand; presence of whorl pattern in both right and left middle finger; presence of simple whorl in left ring finger; tented arch in right little finger and increased finger ridge count in middle finger in both hands; and higher total finger ridge count in left hand when compared to the right hand. Flush terminal plane in children showed absence of arch pattern in ring finger of left hand; presence of loop pattern or whorl pattern in the index finger of both hands; presence of simple whorl in left ring finger; absence of tented arch in left ring finger and right little finger; and increased ridge count in right index and little finger.

The results of the present study provided an insight into specific dermatoglyphic patterns which could be used as potential anatomical tool for predicting the future terminal plane of the primary dentition. This could help the operator to establish necessary measures to ensure no further loss of space occurs which could affect the developing occlusion. However, we acknowledge that further studies with large samples are required to shed more light on this relationship, and if this association between them is proved on larger scale, no wonder it will be a very good marker to predict the developing malocclusion which can be prevented, intercepted or guided to achieve ideal occlusion.

## Conclusions

Within the limitations of the current study, dermatoglyphic patterns can aid in predicting future malocclusions in an earlier stage so as to help in space management and preventive orthodontic treatments.
